# Long-term effectiveness of the trabecular micro-bypass (iStent): 3-year real world data in glaucoma and ocular hypertension

**DOI:** 10.1007/s10792-025-03848-0

**Published:** 2025-11-24

**Authors:** Stafford Sansome, Ujjwal Banerjee, Benjamin Griffin, Sara Issa, Cristina Ginés-Gallego, Madalina Pavel, Mo Abu-Bakra, Sameer Trikha, Avi Kulkarni, Gerassimos Lascaratos, Obeda Kailani

**Affiliations:** 1 King’s College Hospital NHS Foundation Trust, London, UK; 2 Queen Mary Hospital Sidcup, King’s College Hospital NHS Foundation Trust, London, UK; 3 Wellcome Sanger Institute, Wellcome Trust Genome Campus, Saffron Walden, Hinxton, UK; 4 Department of Ophthalmology, Al-Balqa Applied University, As-Salt, Jordan; 5 Faculty of Life Sciences & Medicine, King’s College London, London, SE1 7EH UK

**Keywords:** Glaucoma, Microinvasive glaucoma surgery (MIGS), iStent, Cataract, Phacoemulsification

## Abstract

**Purpose:**

This study evaluates the effectiveness and safety profile of the iStent inject® combined with cataract surgery, with 3 years of follow-up in a large patient cohort (n = 464) of various glaucoma phenotypes and disease severity.

**Methods:**

This retrospective, single-arm, multi-surgeon study included eyes undergoing iStent inject® with cataract surgery. Inclusion criteria was symptomatic cataract in addition to uncontrolled intraocular pressure (IOP) on ≥ 2 IOP lowering agents or contraindication and/or intolerance to IOP lowering medication. IOP, medications, safety profile and cumulative success rates were assessed.

**Results:**

The mean IOP was 14.8 ± 4.5 mmHg at 3 years, compared to 18.3 ± 5.8 mmHg preoperatively, an IOP reduction of 19.1% (*P* < 0.001). The mean number of IOP lowering medications was 1.84 ± 1.40 at 3 years, compared to 2.49 ± 1.12 medications preoperatively. At 3 years 24% of eyes were medication free compared to 3% of eyes preoperatively (*P* < 0.001). At 3 years the cumulative probability of eyes achieving complete success; an IOP reduction of ≥ 20% and IOP 6–21 mmHg without medication was 37% and the cumulative probability of achieving this with medication (qualified success) was 67%. No cases of endophthalmitis, hypotony or other sight threatening complications were recorded. 5% of eyes required further glaucoma procedures.

**Conclusions:**

This large, real-world cohort demonstrates a significant and sustained IOP reduction with an improvement in medication burden 3 years after iStent inject® and phacoemulsification. An excellent safety profile was displayed with a low rate of further glaucoma surgery required.

## Introduction

The burden of glaucoma on the global population is well known, with primary open angle glaucoma (POAG) the leading cause of irreversible blindness worldwide [1]. As global life expectancy continues to increase, so does the prevalence of glaucoma and the need to optimise management of these patients to preserve vision throughout their lifetime [2]. Management of glaucoma is focussed on intraocular pressure (IOP) control with topical medications, laser treatment and filtration surgery the traditional measures used to lower IOP [3]. The advent of microinvasive glaucoma surgery (MIGS) has created another modality for the treatment of glaucoma patients [4]. Although more modest IOP reductions are typically achieved with MIGS procedures than filtration surgery, they have a significantly lower risk profile [4–11]. They can also be used as an alternative or adjunct to topical medication or employed before escalating to filtration surgery following a consideration of the risk–benefit profile for a given patient.

The iStent inject® (Glaukos, San Clemente, CA, USA) is a trabecular micro-bypass stent, the original version (iStent®) was the first MIGS device receiving approval for use in 2012 [12]. It can be used as a standalone device or combined with cataract surgery; two heparin coated titanium stents are injected directly through the trabecular meshwork under direct visualisation with a gonioscopy lens [12]. There have been numerous publications to date which have demonstrated the effectiveness of iStent inject® and iStent® in addition to favourable safety profiles in primary open angle glaucoma and ocular hypertension (OHT) [5–11, 13–19].

In this study, we provide data on the effectiveness and safety profile of iStent inject® combined with cataract surgery, with 3 years of postoperative follow-up in a large patient cohort.

## Methods

### Study design and participants

This was a retrospective, single-arm, multi-centre, multi-surgeon study of eyes that underwent iStent inject® implantation combined with phacoemulsification and intraocular lens (IOL) implantation, evaluating the clinical effectiveness and safety profile in various glaucoma phenotypes and OHT. Data was collected over a 6-year period across a single NHS Foundation Trust from surgery performed at 2 sites, between September 2017 and July 2023.

Inclusion criteria were symptomatic cataract in addition to uncontrolled IOP on ≥ 2 IOP lowering agents or contraindication and/or intolerance to IOP lowering medication. Uncontrolled IOP was defined by that above clinician set individual IOP targets according to disease severity. Patients required a diagnosis of a subtype of glaucoma, primary angle closure (PAC), OHT or be considered a glaucoma suspect (as defined by European Glaucoma Society Terminology and Guidelines for Glaucoma) [20]. Patients were excluded if they had undergone previous invasive glaucoma surgery (trabeculectomy, aqueous tube or shunt or any form of MIGS). Patients with uveitic, neovascular or traumatic glaucoma were excluded, due to abnormal angle morphology which precludes insertion of iStent inject® into the trabecular meshwork. Additional exclusion criteria included any patient undergoing an additional procedure at the time of cataract and iStent implantation such as endoscopic cyclophotocoagulation (ECP) or goniosynechialysis (GSL). Patients with less than 12-month postoperative follow-up data were also excluded. The observation period was ended for any patient undergoing filtration surgery or cyclodiode laser after cataract surgery with iStent; no further IOP or medication data was recorded after this time.

### Surgical device and implantation technique

All iStents were performed by 5 different glaucoma consultants or their senior fellows via a temporal approach following phacoemulsification. The nasal angle was visualised using a direct gonioscopy lens and two iStents were inserted into the nasal trabecular meshwork approximately 2 clock hours apart. Postoperatively, IOP lowering medications were modified at the surgeon’s discretion following evaluation of pre-operative IOP and disease severity. Follow up was arranged between one and four weeks post-operatively.

### Data analysis

Data was obtained from the hospital electronic patient records (Medisoft Ltd, Leeds, United Kingdom) and identifiable data were anonymised and categorised in Excel (Microsoft, Washington, USA) spreadsheets. The study was performed in line with the principles of the Declaration of Helsinki. Statistical analysis was done with base R statistics functionality using the “tidyverse” package [21]. Survival analysis was done using the “survival” package [22]. *P*-values were calculated using paired t-test with Bonferroni correction (due to different sample sizes). Results were considered statistically significant for *p*-values < 0.05. Complete and qualified success rates were determined by the proportion of eyes achieving ≥ 20% IOP reduction and an IOP of 6–21 mmHg after surgery without or with medication, respectively.

## Results

### Study population and baseline characteristics

A total of 464 eyes underwent iStent inject® combined with cataract surgery in this retrospective cohort, each with a minimum of 12 months follow up data. Preoperative demographics and ocular characteristics are displayed in Table 1. The mean age at the time of surgery was 77.5 ± 8.7 years with 54.1% of patients being female. Baseline IOP was 18.3 ± 5.8 mmHg with the mean number of IOP lowering medications at 2.49 ± 1.12. Preoperatively 3% of eyes were medication free. Baseline BCVA was 0.38 LogMAR and mean deviation (MD) of visual field − 7.68 ± 8.36 dB. The most common diagnosis was POAG (63%) followed by PACG (14%) and OHT (11%).

**Table 1:** Baseline demographics

	N = 464
*Age (Years)*	
Mean ± SD	77.5 ± 8.7
Range	45–97
*Gender (%)*	
Female	54.1%
*Eye (%)*	
OD	48.1%
*Mean IOP at baseline (mmHg)*	
Mean ± SD	18.3 ± 5.8
95% CI	17.7–18.8
Range	8–41
*BCVA at baseline (LogMAR)*	
Mean	0.38
95% CI	0.34–0.43
Range	− 0.20–2.00
*Number of IOP lowering agents at Baseline*	
Mean ± SD	2.49 ± 1.12
95% CI	2.39–2.59
Range	0–4
*Mean deviation at baseline (dB)*	
Mean ± SD	− 7.68 ± 8.36
*Diagnosis (%)*	
Primary open angle glaucoma	63.0%
Primary angle closure glaucoma	14.0%
Ocular hypertension	11.0%
Glaucoma suspect	3.5%
Primary angle closure	3.3%
Normal tension glaucoma	3.0%
Pseudoexfoliative glaucoma	1.3%
Pigmentary glaucoma	0.9%

SD, Standard deviation; CI, Confidence interval; OD, Left eye; IOP, Intraocular pressure; mmHg, Millimetres of mercury; BCVA, Best corrected visual acuity; dB, Decibels

### Intraocular pressure and medication use

**Fig. 1:**
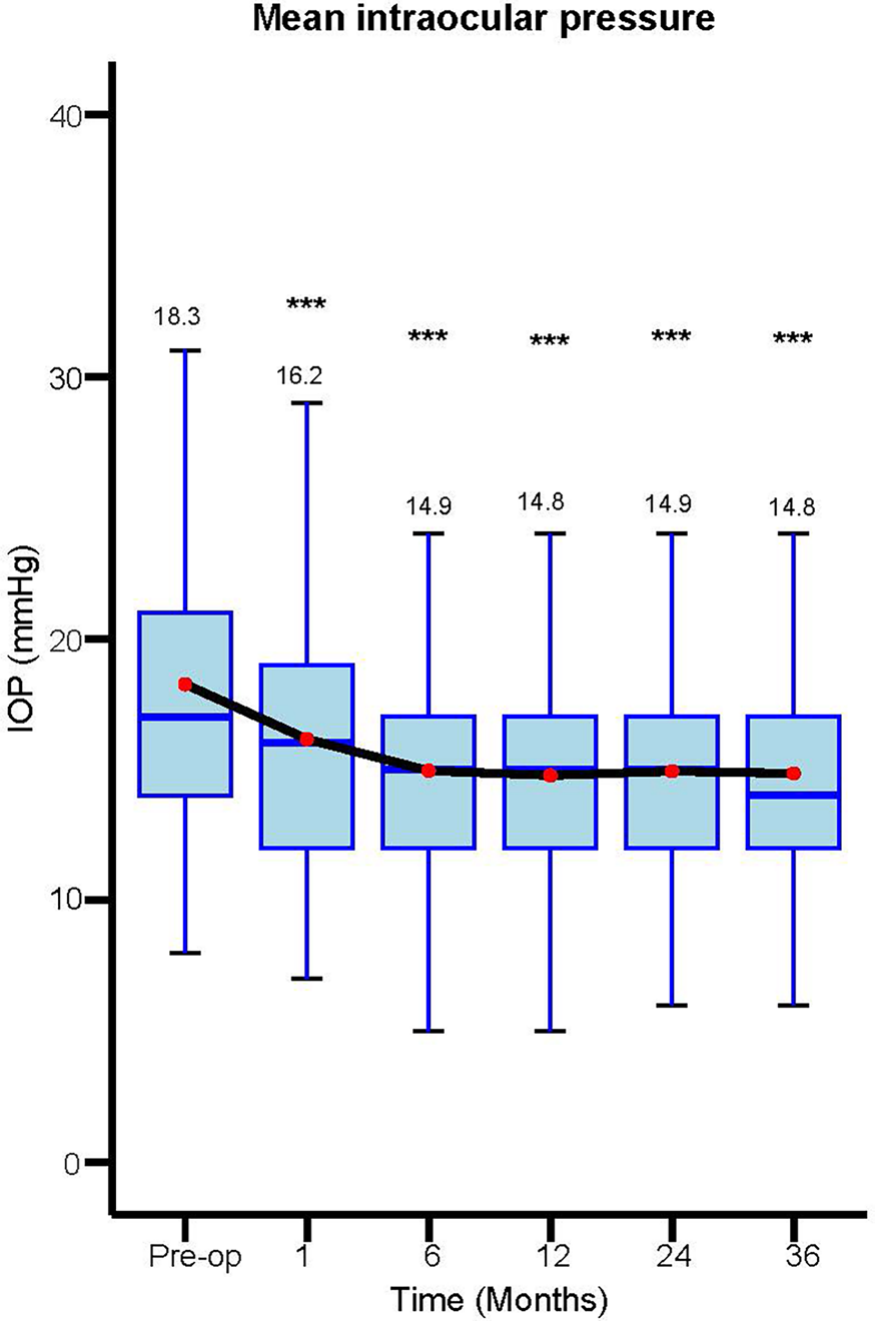
Mean intraocular pressure over time. Pre-op n = 464, 1 month n = 418, 6 months n = 460, 12 months n = 461, 24 months n = 461, 36 months n = 175. Abbreviations: *** = *p* value < 0.001, IOP = intraocular pressure

There was a statistically significant reduction in IOP at all time points postoperatively (*P* < 0.001). The mean IOP was 14.8 ± 4.5 mmHg at 3 years follow up, compared to 18.3 ± 5.8 mmHg preoperatively, representing an IOP reduction of 19.1%. Figure 1 provides mean IOP data at various timepoints up to 3 years of follow up.

**Fig. 2:**
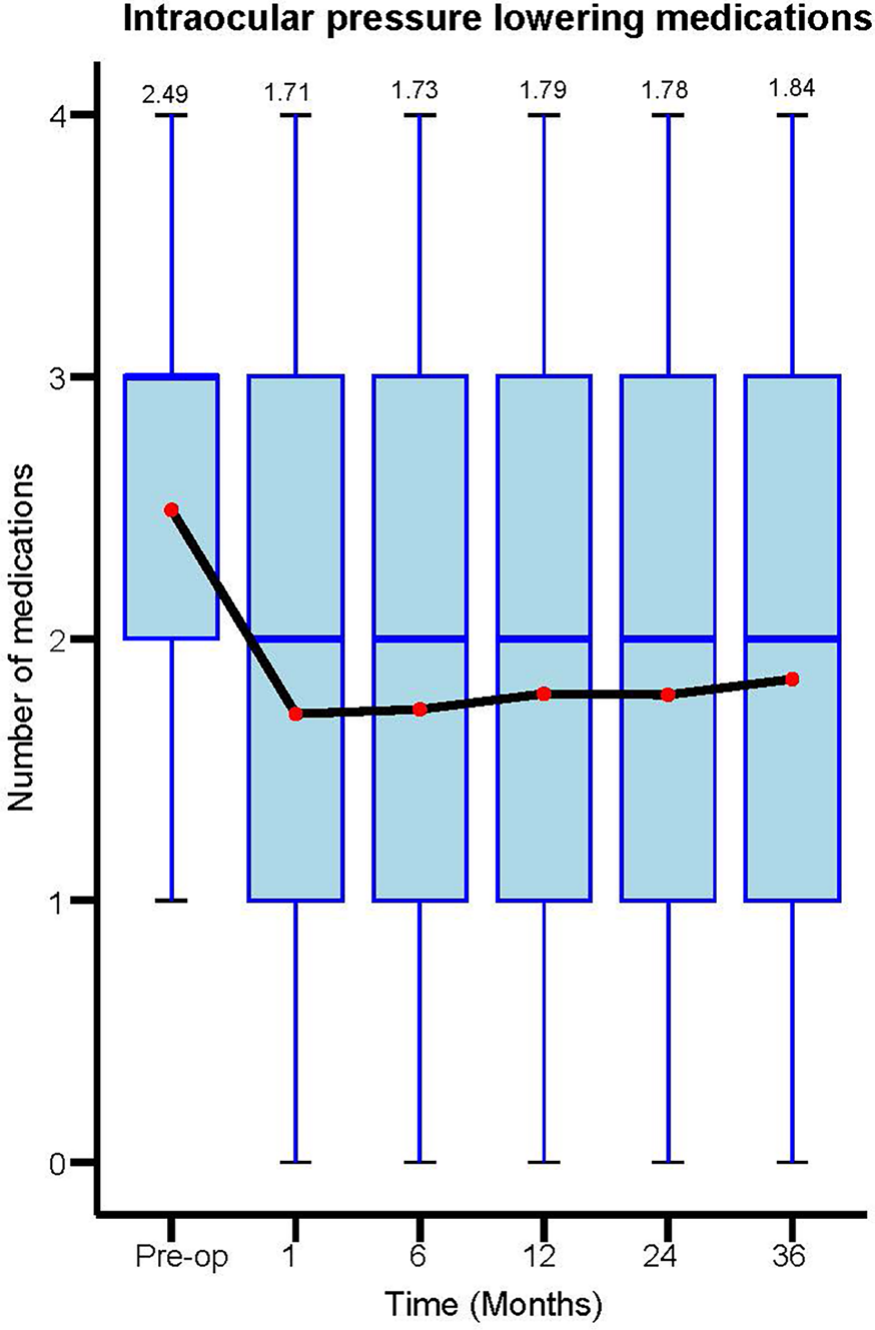
Mean number of intraocular pressure (IOP) lowering medications over time. Pre-op n = 464, 1 month n = 455, 6 months n = 458, 12 months n = 464, 24 months n = 265, 36 months n = 129

**Fig. 3:**
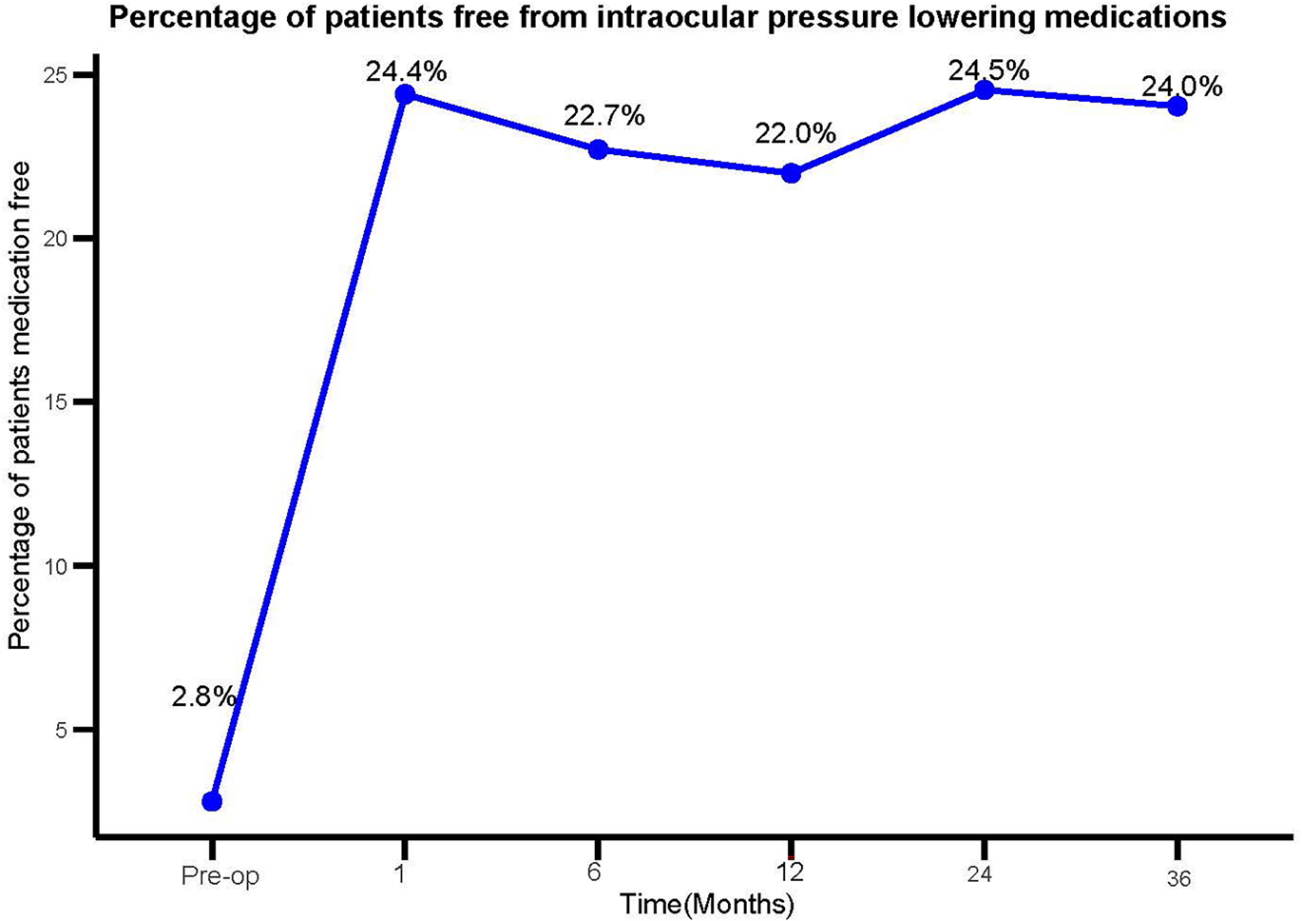
Percentage of patients free from intraocular pressure lowering medications over time. Pre-op n = 464, 1 month n = 455, 6 months n = 458, 12 months n = 464, 24 months n = 265, 36 months n = 129

There was a statistically significant reduction in the number of IOP lowering medications at all time points postoperatively (*P* < 0.001). The mean number of IOP lowering medications was 1.84 ± 1.40 at 3 years follow up, compared to 2.49 ± 1.12 medications preoperatively, representing a mean reduction of 0.65 medications per eye. Figure 2 provides data on the mean number of IOP lowering medications at various timepoints up to 3 years of follow up. At 3 years 24% of eyes were medication free compared to 3% of eyes preoperatively (*P* < 0.001). The percentage of eyes that remained medication free was statistically significant at all time points of follow up which is outlined in Fig. 3.

### Success rates

**Fig. 4:**
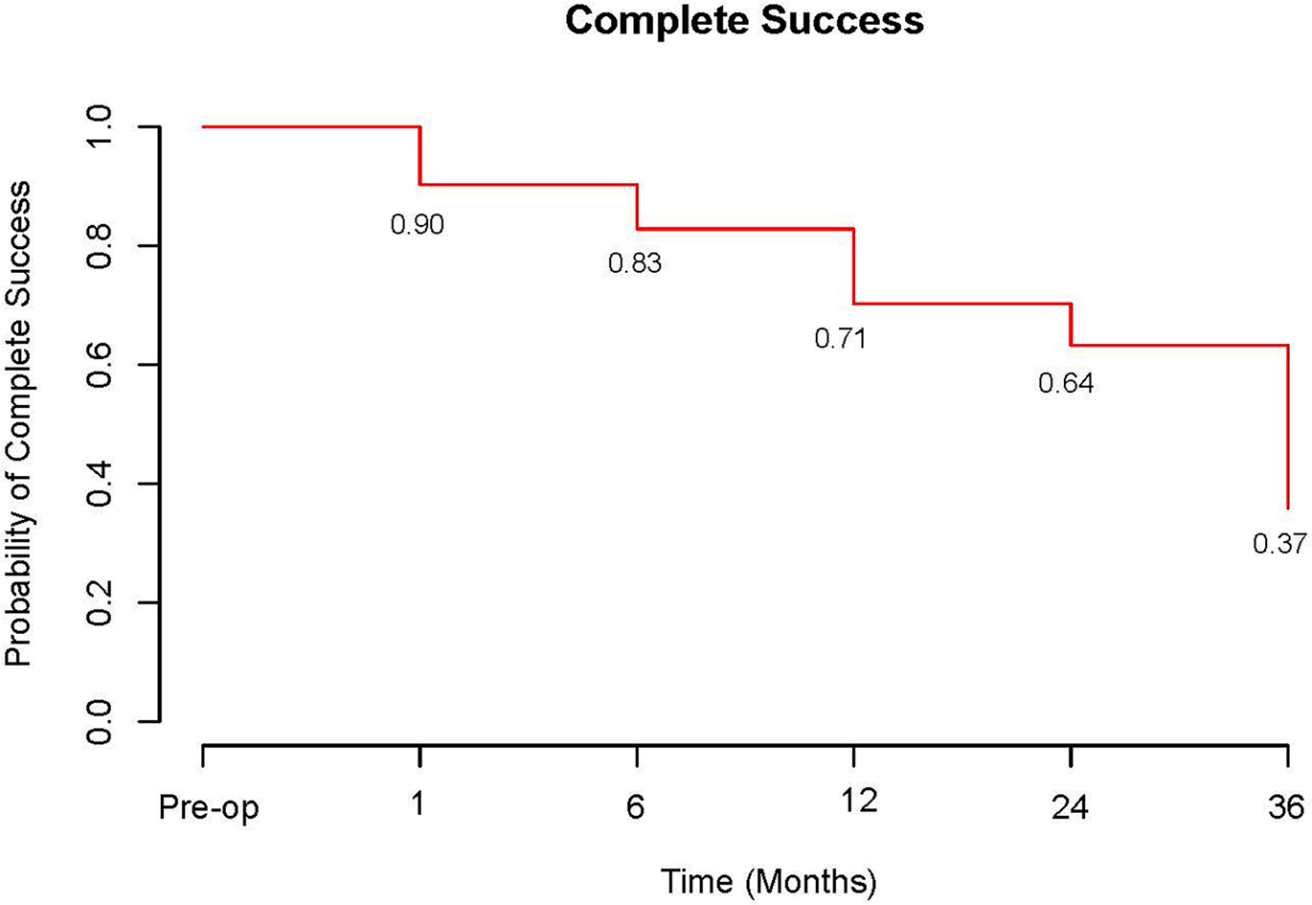
Complete success over time. Defined as an intraocular pressure reduction of ≥ 20% and IOP 6–21 mmHg without medication

**Fig. 5:**
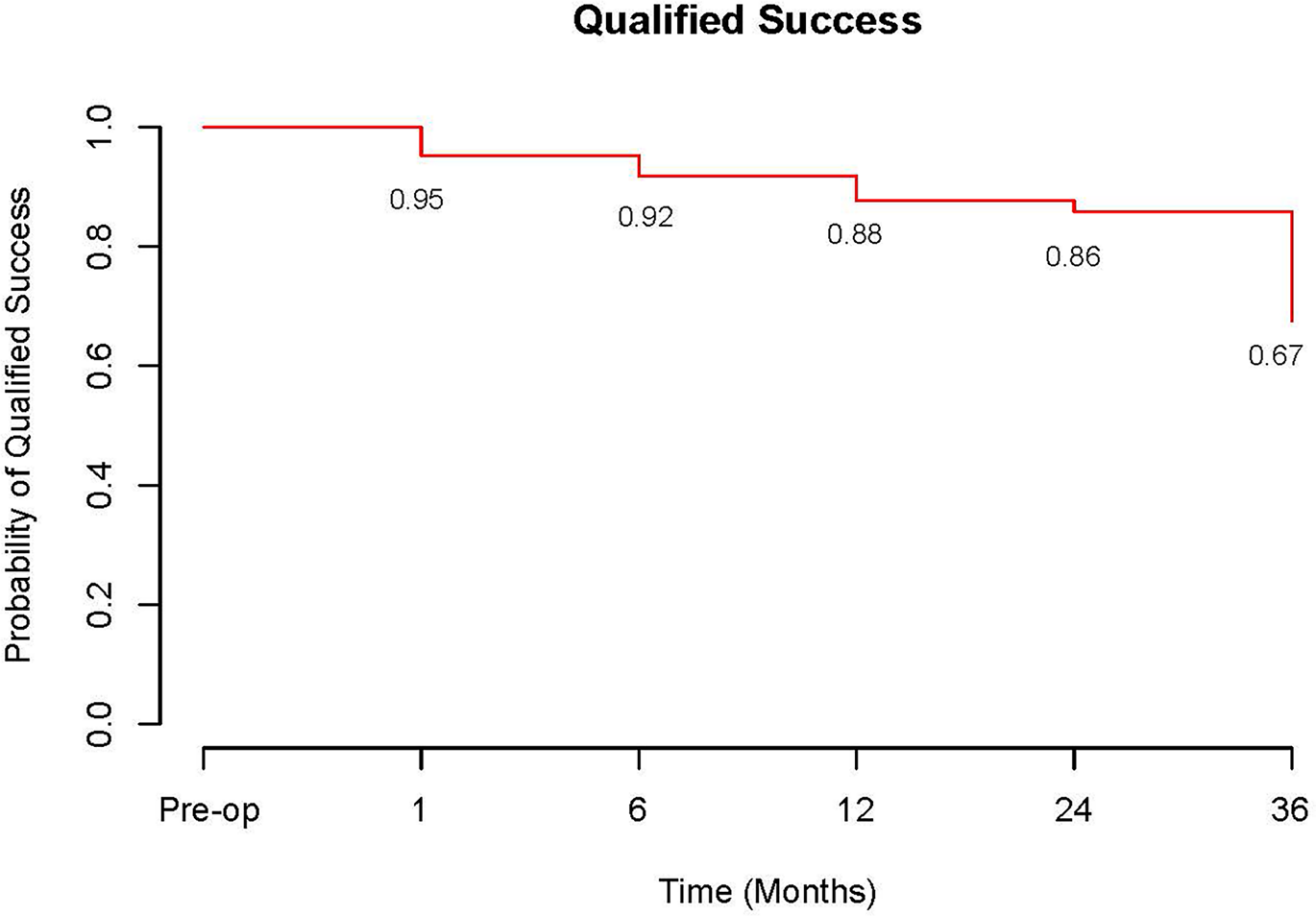
Qualified success over time. Defined as an intraocular pressure reduction of ≥ 20% and IOP 6–21 mmHg with medication

At 3 years follow up the cumulative probability of eyes achieving complete success; an IOP reduction of ≥ 20% and IOP 6–21 mmHg without medication was 37% (Fig. 4). Cumulative probability of achieving this with medication (qualified success) was 67% (Fig. 5). The probability of complete success was 71% at 1 year and 64% at 2 years compared to the probability of qualified success which was 88% at 1 year and 86% at 2 years.

### Subgroup analysis

Subgroup analysis was also performed. Severity of glaucoma classified by early (MD > − 6D), moderate (MD − 6 to − 12 dB) and severe (MD < − 12 dB) showed no statistically significant difference between these groups with regards to IOP or medication reduction across the various timepoints.

Comparison of IOP and medication reduction between glaucoma (POAG/NTG) and glaucoma suspects and OHT subgroups was performed (with data up to 2 years given small dataset available for OHT and glaucoma suspect patients at 3 years follow up). The mean IOP reduction in glaucoma eyes was 15.9% at 2 years (14.8 ± 4.56 mmHg at 2 years vs 17.6 ± 5.22 mmHg preoperatively) with a mean IOP reduction in glaucoma suspect and OHT eyes of 15.7% at 2 years (15.6 ± 3.76 mmHg at 2 years vs 18.5 ± 5.17 mmHg preoperatively). There was a statistically significant difference in reduction of IOP lowering medications between glaucoma and glaucoma suspect and OHT eyes at 2 years (*P* < 0.05). The mean number of IOP lowering medications for glaucoma eyes were reduced by 23.4% (0.61 drops) at 2 years (2.00 ± 1.32 at 2 years vs 2.61 ± 1.12 preoperatively) with medications for glaucoma suspect and OHT eyes reduced by 34.5% (0.69 drops) at 2 years (1.31 ± 1.26 at 2 years vs 2.00 ± 1.13 preoperatively). At 2 years 18% of glaucoma eyes were medication free (2% preoperatively) compared to 39% of glaucoma suspect and OHT eyes that were medication free at 2 years (6% preoperatively) (*p* < 0.05).

### Safety data

There was a statistically significant post-operative improvement in BCVA at all time points before 3 years. At 12 moths BCVA was 0.19 compared to 0.38 preoperatively. Vision loss equivalent to ≥ 2 lines on Snellen occurred in 0.9% of eyes (4/464). Intra-operative complications recorded were hyphaema 0.6% (3/464), iris trauma or prolapse 0.6% (3/464), vitreous loss 0.4% (2/464), endothelial damage 0.4% (2/464) and phacoemulsification wound burn 0.2% (1/464). Post-operative complications recorded were uveitis 3.7% (17/464), macular oedema 2.2% (10/464), corneal oedema 1.7% (8/464), hyphaema 0.9% (4/464), iris to wound 0.2% (1/464) and wound leak 0.2% (1/464). One eye required an anterior chamber washout due to a persistent hyphaema with a raised IOP. No cases of endophthalmitis or hypotony were recorded. There were no cases of corneal decompensation recorded at any time point. A total of 5% of eyes (28/464) required further glaucoma procedures. Preserflo MicroShunt was subsequently performed in 2.2% of eyes (10/464), Trabeculectomy in 1.3% of eyes (6/464), Selective Laser Trabeculoplasty (SLT) in 1.1% (5/464) and cyclodiode laser in 0.4% (2/464).

## Discussion

In this retrospective, single-arm, multi-centre, multi-surgeon study we report the effectiveness and safety profile of the iStent inject® combined with cataract surgery in a large cohort with 3 years follow up data. These 464 eyes include various glaucoma subtypes and severity of glaucoma, along with a high pre-operative medication burden. A significant and sustained reduction in IOP along with a reduction in IOP lowering medication was demonstrated throughout the 3 years of post-operative follow up, with favourable safety data.

The baseline characteristics of this study’s population were heterogenous with regards to disease severity, baseline IOP and medication burden as well as the subtype of glaucoma. There was a relatively high baseline IOP (18.3 mmHg), high pre-operative medication burden (2.49 medications) and significant degree of baseline visual field loss (MD − 7.68 Db). Inclusion criteria were broad and there were few indications for exclusion, in contrast to many studies in the literature which have narrow and pre-defined inclusion criteria. Therefore, this provides clinically relevant data reflective of a diverse glaucoma population with significant disease burden which can help to inform patients and surgeons with regards to evaluating glaucoma treatment options.

Our cohort achieved a statistically significant reduction in IOP maintained at 3 years follow up with an average reduction of 19.1% (from a baseline of 18.3–14.8 mmHg at 3 years). This was in addition to a 0.65 reduction in use of IOP lowering medications per eye (1.84 at 3 years vs 2.49 at baseline). At 3 years 24% of eyes were medication free compared to 3% of eyes preoperatively, which represents a sevenfold increase from baseline. These outcomes align to those of a 3 year multicentre, retrospective study of 273 eyes by Clement et al. [10]. This included various glaucoma subtypes or OHT with eyes undergoing iStent inject® combined with cataract surgery. There was a mean IOP reduction in this cohort of 15.5% (from a baseline of 16.4–13.9 mmHg at 3 years) and a 1.03 reduction in use of IOP lowering medications per eye (0.48 at 3 years vs 1.51 at baseline). At 3-years 71% of eyes were medication-free (versus 21.6% pre-operatively). Another comparable study was carried out by Salimi et al. who published 3 year outcomes of 124 eyes with various glaucoma subtypes undergoing iStent inject® combined with cataract surgery [11]. This demonstrated a 22% reduction in IOP from 16.9 mmHg at baseline to 13.2 mmHg at 3 years, along with a decrease in IOP medication burden of 1.22 medications (1.16 at 3 years vs 2.38 at baseline). Liu et al. also published 3 year outcomes of 150 eyes with open angle glaucoma and OHT who underwent iStent inject® combined with cataract surgery [9]. A significant reduction in IOP was demonstrated at all time points with IOP reducing by 8.7% at 3 years (16.98 mmHg at baseline vs 15.51 mmHg at 3 years) but there was no significant reduction in IOP lowering medication at 3 years form a baseline of 2.54 medications (2.76 medications at 3 years).

The outcomes we demonstrate in our show a considerable reduction in IOP of 19.1% at 3 years which compares favourably to that of many other studies in the literature. This may in part be due to a relatively high baseline IOP of 18.3 mmHg, however our cohort also included patients with normal tension glaucoma (NTG) which would conversely impact the postoperative reduction in IOP that could be expected to be achieved. The relatively high baseline IOP indicates a greater disease burden in our cohort which is likely more reflective of the realities of day-to-day clinical practice compared to studies containing strict inclusion criteria leading to less representative cohorts. Despite our cohort showing a significant reduction in IOP lowering medication use, this was more modest than many other studies. Again, given the higher disease burden of our cohort, it could be expected that IOP lowering medication would be less likely to be discontinued post-operatively, regardless of the surgical outcome, owing to a more advanced glaucoma status requiring a lower IOP target.

The subgroup analysis carried out demonstrated differences in outcomes according to the disease phenotype. Although a comparable IOP reduction was seen in glaucoma (POAG/NTG) eyes compared to glaucoma suspect and OHT eyes (15.9% vs 15.7% respectively), there was a significantly greater reduction in medication burden in glaucoma suspect and OHT eyes compared to glaucoma eyes (34.5% vs 23.4% drop reduction and 39% vs 18% eyes medications free respectively). These findings could represent a tendency for clinicians to be more willing to reduce IOP medications in those without an established glaucoma diagnosis as opposed to those with established glaucoma who would have a lower target IOP. Despite this, similar IOP reductions were obtained and therefore it is possible that eyes without established glaucoma may obtain greater benefit from iStent in the presence of a ‘healthier’ trabecular meshwork and Schlemm’s canal. This is an area that warrants further investigation.

Favourable safety data from our cohort is in keeping with that of existing studies [5–11, 13–19]. An improvement in BCVA was seen in the first 2 years post-operatively (maintained thereafter), recorded minor intra-operative and post-operative complications were only marginally above the incidence seen in cataract surgery alone as per the National Ophthalmology Database audit [23]. Filtration surgery for glaucoma was subsequently required in 3.5% of eyes over 3 years (trabeculectomy or Preserflo MicroShunt). There are few studies on the rates of patients undergoing filtration surgery for glaucoma, those available demonstrate rates of 4.2% at 4 years and 5.3% at 5 years in patients newly diagnosed with POAG [24–26]. These are relatively aligned to our findings especially given many patients within our study likely had long established glaucoma diagnoses.

Despite their established safety and efficacy, IOP lowering medications have numerous drawbacks, specifically their dependence of patient adherence, local adverse effects and impact upon patients’ quality of life [27–30]. Studies have also demonstrated that a higher glaucoma medication intensity is a predictor for failure of glaucoma filtration surgery [31]. Consequently, our findings of a reduced medication burden and increase in medication free eyes post-surgery, demonstrate clear potential benefits for glaucoma patients who can achieve a given IOP with fewer potential side effects and impact upon their quality of life from their glaucoma medications in addition to possible improved outcomes if subsequent filtration surgery is required. Furthermore, many glaucoma services are shifting towards providing more ‘virtual’ clinic appointments to provide greater capacity, with recent research indicating comparable running costs to face to face clinics [32]. Medication free patients post iStent inject® combined with cataract surgery would be good candidates for ongoing review in these clinics and therefore have the potential to maximise capacity and provide benefits for service delivery. Further research is therefore warranted to establish if the use of iStent inject® combined with phacoemulsification translates in better long-term quality of life outcomes for patients, in addition to service delivery benefits for glaucoma departments.

We acknowledge that there are several limitations to our study. The absence of a control group restricts comparison between the effects of iStent inject® and that of cataract surgery alone. Studies have shown that cataract surgery alone can lower IOP by greater than 20% [33–35], however this effect wears off over time with studies demonstrating a reduction in the IOP lowering effect of cataract surgery alone as early as 12 months post-operatively [33, 34, 36]. Our study demonstrates a sustained reduction in IOP at 3 years which indicates iStent inject® may have a role in sustaining IOP reductions beyond that of cataract surgery alone. Furthermore, studies directly comparing combined iStent and cataract surgery to that of cataract surgery alone have shown greater reductions in IOP and reduced medication burden in those undergoing iStent combined with cataract surgery [14–16]. The efficacy of iStent inject alone has also been demonstrated in a recent 7 year prospective study with similar results to that of iStent inject combined with cataract surgery [8].

Another limitation is that no pre-operative medication washout was performed (of IOP lowering medications), which could potentially decrease the IOP lowering effect and reduction in medication burden post-operatively. However, real-world practice often necessitates the continuation of IOP lowering medication, given the risk of progression especially in patients with moderate to advanced glaucoma whilst awaiting surgery and in the immediate post-operative period. Although no cases of corneal decompensation were recorded, corneal endothelial cell counts were not measured pre and post operatively. This is an area that requires more research to understand the impact of angle surgery such as iStent inject® on the cornea (a protocol has now been introduced in our institution to measure pre and post operative endothelial cell counts for all patients undergoing MIGS or filtration surgery). Finally, the cohort of patients followed up to 3 years was significantly smaller than earlier time points. With healthcare service pressures and limited appointment availability there is a possibility longer follow up is biased towards patients with suboptimal IOP control as opposed to stable patients who require less frequent or even community follow up.

## Conclusions

This large, real-world cohort demonstrates a significant and sustained IOP reduction with a marked improvement in medication burden 3 years after combined iStent inject® implantation and phacoemulsification. An excellent safety profile was displayed with a low rate of further glaucoma surgery required. These findings were demonstrated in a diverse patient cohort including both open and closed angle glaucoma with varying disease severity and OHT. Further research is warranted to establish if the use of iStent inject® combined with phacoemulsification translates in better long-term quality of life outcomes for patients, in addition to service delivery benefits for glaucoma departments.

## Data Availability

Available on request.

## References

[1] Rawal L, GBD 2019 Blindness Causes Collaborators (2021) Causes of blindness and vision impairment in 2020 and trends over 30 years, and prevalence of avoidable blindness in relation to VISION 2020: The Right to Sight: an analysis for the Global Burden of Disease Study.

[2] Allison K, Patel D, Alabi O (2020) Epidemiology of glaucoma: the past, present, and predictions for the future. Cureus 12(11):11686 10.7759/cureus.11686 PMC7769798 33391921

[3] Weinreb RN, Aung T, Medeiros FA (2014) The pathophysiology and treatment of glaucoma: a review. JAMA 311(18):1901–1911 24825645 10.1001/jama.2014.3192 PMC4523637

[4] Saheb H, Ahmed IIK (2012) Micro-invasive glaucoma surgery: current perspectives and future directions. Curr Opin Ophthalmol 23(2):96–104 22249233 10.1097/ICU.0b013e32834ff1e7

[5] Berdahl J, Voskanyan L, Myers JS, Katz LJ, Samuelson TW (2020) iStent inject trabecular micro‐bypass stents with topical prostaglandin as standalone treatment for open‐angle glaucoma: 4‐year outcomes. Clin Exp Ophthalmol 48(6):767–774 32311201 10.1111/ceo.13763

[6] Neuhann TH, Hornbeak DM, Neuhann RT, Giamporcaro JE (2019) Long-term effectiveness and safety of trabecular microbypass stent implantation with cataract surgery in patients with glaucoma or ocular hypertension: five-year outcomes. J Cataract Refract Surg 45(3):312–320 30851807 10.1016/j.jcrs.2018.10.029

[7] Arriola-Villalobos P, Martinez-de-la-Casa JM, Diaz-Valle D, Morales-Fernandez L, Fernandez-Perez C, Garcia-Feijoo J (2016) Glaukos iStent inject® trabecular micro-bypass implantation associated with cataract surgery in patients with coexisting cataract and open-angle glaucoma or ocular hypertension: a long-term study. J Ophthalmol. 10.1155/2016/1056573 27882243 10.1155/2016/1056573 PMC5108856

[8] Hengerer FH, Auffarth GU, Conrad-Hengerer I (2024) 7-Year efficacy and safety of iStent inject trabecular micro-bypass in combined and standalone usage. Adv Ther. 10.1007/s12325-024-02788-y 38363465 10.1007/s12325-024-02788-y PMC10960914

[9] Liu J, Bouhout S, Massicotte E, Desgroseilliers A, Lord F (2023) Three-year ef-ficacy and safety outcomes of the second-generation trabecular micro-bypass stents in conjunction with cataract surgery in ocular hypertension and open-angle glaucoma. J Ophthalmol Vis Sci 8(4):1087

[10] Clement C, Howes F, Ioannidis A, Shiu M, Manning D, Lusthaus JA, Goodwin TW (2022) Multicenter effectiveness and disease stability through 3 years after istenttrabecular micro-bypass with phacoemulsification in glaucoma and ocular hypertension. Clin Ophthalmol (Auckland, NZ) 16:2955 10.2147/OPTH.S373290 PMC9444145 36071724

[11] Salimi A, Watt H, Harasymowycz P (2021) Three-year outcomes of second-generation trabecular micro-bypass stents (iStent inject) with phacoemulsification in various glaucoma subtypes and severities. J Glaucoma 30(3):266–275 33105306 10.1097/IJG.0000000000001716

[12] Bahler CK, Hann CR, Fjield T, Haffner D, Heitzmann H, Fautsch MP (2012) Second-generation trabecular meshwork bypass stent (iStent inject) increases outflow facility in cultured human anterior segments. Am J Ophthalmol 153(6):1206–1213 22464365 10.1016/j.ajo.2011.12.017

[13] Holmes DP, Clement CI, Nguyen V, Healey PR, Lim R, White A, Yuen J, Lawlor M (2022) Comparative study of 2‐year outcomes for Hydrus or iStent inject microinvasive glaucoma surgery implants with cataract surgery. Clin Exp Ophthalmol 50(3):303–311 35077009 10.1111/ceo.14048

[14] Samuelson TW, Sarkisian SR Jr, Lubeck DM, Stiles MC, Duh YJ, Romo EA, Cotter F (2019) Prospective, randomized, controlled pivotal trial of an ab interno implanted trabecular micro-bypass in primary open-angle glaucoma and cataract: two-year results. Ophthalmology 126(6):811–821 30880108 10.1016/j.ophtha.2019.03.006

[15] Salimi A, Abu-Nada M, Harasymowycz P (2021) Matched cohort study of cataract surgery with and without trabecular microbypass stent implantation in primary angle-closure glaucoma. Am J Ophthalmol 224:310–320 33428885 10.1016/j.ajo.2020.12.032

[16] Chen DZ, Sng CC, Sangtam T, Thomas A, Shen L, Huang PK, Cheng J (2020) Phacoemulsification vs phacoemulsification with micro‐bypass stent implantation in primary angle closure and primary angle closure glaucoma: a randomized single‐masked clinical study. Clin Exp Ophthalmol 48(4):450–461 32003538 10.1111/ceo.13721

[17] Singh IP, Sarkisian S, Hornbeak D, Katz LJ, Samuelson T, iStent inject Study Group (2021) Treatment success across different levels of preoperative disease burden: stratified two-year outcomes from the pivotal trial of iStent inject® trabecular micro-bypass in primary open-angle glaucoma and cataract. Clin Ophthalmol. 10.2147/OPTH.S316270 34376967 10.2147/OPTH.S316270 PMC8349204

[18] Hengerer FH, Auffarth GU, Conrad-Hengerer I (2022) iStent inject trabecular micro-bypass with or without cataract surgery yields sustained 5-year glaucoma control. Adv Ther 39(3):1417–1431 35113323 10.1007/s12325-021-02039-4 PMC8918186

[19] Lindstrom R, Sarkisian SR, Lewis R, Hovanesian J, Voskanyan L (2020) Four-year outcomes of two second-generation trabecular micro-bypass stents in patients with open-angle glaucoma on one medication. Clin Ophthalmol. 10.2147/OPTH.S235293 32021070 10.2147/OPTH.S235293 PMC6968820

[20] Pazos M, Traverso CE, Viswanathan A (2025) European glaucoma society-terminology and guidelines for glaucoma. Br J Ophthalmol 109(Suppl 1):1–212 41026937 10.1136/bjophthalmol-2025-egsguidelines

[21] Wickham H, Averick M, Bryan J, Chang W, McGowan LD, François R, Grolemund G, Hayes A, Henry L, Hester J (2019) Welcome to the tidyverse. J Open Source Softw 4(43):1686

[22] Therneau T (2015) A package for survival analysis in S. R Packag Vers 2(7):2014

[23] National Ophthalmology Database Audit. Year 7 Annual Report—The Sixth Prospective Report of the National Ophthalmology Database Audit National Cataract Audit

[24] Lee SJ, Lee SA, Lee S, Bae HW, Kim CY, Seong GJ, Park JW, Lee K (2022) Risk factors for undergoing surgery in patients with newly diagnosed open-angle glaucoma. Sci Rep 12(1):5661 35383265 10.1038/s41598-022-09832-3 PMC8983768

[25] Szigiato A, Trope GE, Jin Y, Buys YM (2015) Trends in glaucoma surgical procedures in Ontario: 1992–2012. Can J Ophthalmol 50(5):338–344 26455967 10.1016/j.jcjo.2015.07.005

[26] Schwartz GF, Patel A, Naik R, Lunacsek O, Ogbonnaya A, Campbell J (2021) Characteristics and treatment patterns of newly diagnosed open-angle glaucoma patients in the United States: an administrative database analysis. Ophthalmol Glaucoma 4(2):117–125 32927109 10.1016/j.ogla.2020.09.002

[27] Skalicky SE, Goldberg I, McCluskey P (2012) Ocular surface disease and quality of life in patients with glaucoma. Am J Ophthalmol 153(1):1–9 21872203 10.1016/j.ajo.2011.05.033

[28] Beckers HJ, Schouten JS, Webers CA, van der Valk R, Hendrikse F (2008) Side effects of commonly used glaucoma medications: comparison of tolerability, chance of discontinuation, and patient satisfaction. Graefes Arch Clin Exp Ophthalmol 246:1485–1490 18575878 10.1007/s00417-008-0875-7

[29] Romano J, Ferreira N, Godinho G, Tomás R, Oliveira N, Sousa JP, Godinho G Sr (2024) Understanding ocular surface disease in glaucoma: a comparative analysis of symptoms and objective parameters. Cureus 16(2):54070 10.7759/cureus.54070 PMC10936608 38481920

[30] Moore SG, Richter G, Modjtahedi BS (2023) Factors affecting glaucoma medication adherence and interventions to improve adherence: a narrative review. Ophthalmol Ther 12(6):2863–2880 37698824 10.1007/s40123-023-00797-8 PMC10640536

[31] Wong JKW, Leung TK, Lai JS, Chan JC (2022) Evaluation of adverse effects of topical glaucoma medications on trabeculectomy outcomes using the glaucoma medications intensity index. Ophthalmol Ther 11(1):387–401 34932180 10.1007/s40123-021-00447-x PMC8770766

[32] Shah V, Jackson TL, Edwards RT, Attlee J, Kailani O (2024) Health economics of virtual versus face-to-face glaucoma clinics: a time-driven activity-based costing study. BMJ Open Ophthalmol. 10.1136/bmjophth-2024-001800 39613389 10.1136/bmjophth-2024-001800 PMC11605819

[33] Vold S, Ahmed IIK, Craven ER, Mattox C, Stamper R, Packer M, Brown RH, Ianchulev T, CyPass Study Group (2016) Two-year COMPASS trial results: supraciliary microstenting with phacoemulsification in patients with open-angle glaucoma and cataracts. Ophthalmology 123(10):2103–2112 27506486 10.1016/j.ophtha.2016.06.032

[34] Ahmed IIK, De Francesco T, Rhee D, McCabe C, Flowers B, Gazzard G, Samuelson TW, Singh K, HORIZON Investigators (2022) Long-term outcomes from the HORIZON randomized trial for a Schlemm’s canal microstent in combination cataract and glaucoma surgery. Ophthalmology 129(7):742–751 35218867 10.1016/j.ophtha.2022.02.021

[35] Poley BJ, Lindstrom RL, Samuelson TW, Schulze R Jr (2009) Intraocular pressure reduction after phacoemulsification with intraocular lens implantation in glaucomatous and nonglaucomatous eyes: evaluation of a causal relationship between the natural lens and open-angle glaucoma. J Cataract Refract Surg 35(11):1946–1955 19878828 10.1016/j.jcrs.2009.05.061

[36] Armstrong JJ, Wasiuta T, Kiatos E, Malvankar-Mehta M, Hutnik CM (2017) The effects of phacoemulsification on intraocular pressure and topical medication use in patients with glaucoma: a systematic review and meta-analysis of 3-year data. J Glaucoma 26(6):511–522 28333892 10.1097/IJG.0000000000000643

